# Manual and Oscillometric Blood Pressure in tPA‐Treated Acute Ischemic Stroke: What Constitutes Agreement?

**DOI:** 10.1161/SVIN.122.000711

**Published:** 2023-05-24

**Authors:** Mary A. Grove, Mani Paliwal, Anne Shearin, Jane Kaiser, Eun Sun Koo, Danielle Howey, Michele Galati, Bozena Czekalski, Jennifer Dumawal, Briana DeCarvalho, Jackie Dwyer, Georgios Tsivgoulis, Andrei V. Alexandrov, Anne W. Alexandrov

**Affiliations:** ^1^ Hackensack Meridian Health Edison NJ; ^2^ University of Tennessee College of Nursing Memphis TN; ^3^ Ocean University Medical Center Brick NJ; ^4^ Jersey Shore University Medical Center Neptune NJ; ^5^ Southern Ocean Medical Center Manahawkin NJ; ^6^ Riverview Medical Center Red Bank NJ; ^7^ Bayshore Medical Center Holmdel NJ; ^8^ JFK Medical Center Edison NJ; ^9^ National and Kapodistrian University of Athens Athens Greece; ^10^ Banner University Medical Center and University of Arizona Phoenix AZ; ^11^ University of Tennessee Health Science Center Memphis TN

**Keywords:** acute ischemic stroke, alteplase, bland‐altman measures of agreement, blood pressure, limits of agreement, mean arterial pressure, noninvasive oscillometric blood pressure

## Abstract

**Background:**

Automatic noninvasive oscillometric blood pressure (NIBP) devices measure mean arterial pressure (MAP); systolic and diastolic blood pressure (SBP, DBP) are algorithmically derived from MAP. The most invalid NIBP measure is SBP, yet stroke practitioners use it to manage blood pressure (BP) in accordance with thrombolysis guidelines. We determined agreement between SBP, DBP, and MAP measured manually and by NIBP in patients treated with alteplase.

**Methods:**

A multisite prospective observational study of NIBP and manual BP agreement was conducted in patients treated with alteplase immediately after bolus and infusion initiation using methods established in guidelines for the assessment of device agreement. Dual auscultatory stethoscopes were used by 2 investigators to ensure agreement with each manual BP variable and MAP was calculated using the standard formula for manual BP measures. Data were analyzed using Bland–Altman analyses and Lin concordance correlation coefficient.

**Results:**

A total of 7 hospitals participated, collecting 5 sets of manual/NIBP BPs in 95 patients treated with alteplase (475 paired measures). Range in limits of agreement were SBP: −28.91 to 21.41 mmHg with Lin's concordance correlation coefficient 0.8; DBP: −21.0 to 19.0 mmHg with Lin's concordance correlation coefficient 0.69; and MAP: −27.5 to 16.5 mmHg with Lin's concordance correlation coefficient 0.7. There was no difference in device agreement by BP device manufacturer brand. Differences in SBP, DBP, and MAP between NIBP and manual sphygmomanometry failed to reach guideline recommendations requiring 80% of measures to fall within a 5 mmHg difference and 95% of measures to fall within a 10 mmHg difference.

**Conclusion:**

NIBP devices produce significantly different BP measures then manual sphygmomanometry auscultated BP. Because NIBP devices rely on the MAP and do not directly measure SBP and DBP, definition of what constitutes safe MAP boundaries in patients treated with alteplase should be determined when automatic BP measurement is used in clinical practice.

## Graphical abstract

Graphical Abstract

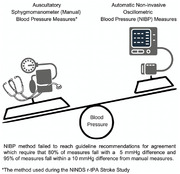

**Nonstandard Abbreviations and Acronyms**
B‐A: Bland–Altman
BHS: British Hypertension Society
ESHIP: European Society of Hypertension International Protocol
NIBP: noninvasive oscillometric blood pressure

**Clinical Perspective**

**What Is New?**

- Noninvasive oscillometric blood pressure devices validly measure only mean arterial pressure, yet the displayed systolic and diastolic blood pressure are commonly used to guide blood pressure management during/after tissue plasminogen activator treatment.

**What Are the Clinical Implications?**

- Differences in systolic blood pressure and diastolic blood pressure between noninvasive oscillometric blood pressure and manual sphygmomanometry fail guideline recommendations requiring 80% of measures to fall within a 5 mmHg difference and 95% of measures to fall within a 10 mmHg difference.
- Safe mean arterial pressure limits for patients treated with alteplase with acute ischemic stroke remain unknown. Our study takes an important first step in reframing the acute ischemic stroke treatment context toward consideration of mean arterial pressure as a key measure to guide the initiation of alteplase treatment and ongoing patient management.

National and international guidelines^1^, ^2^, ^3^, ^4^, ^5^ inform the acceptable limits for systolic blood pressure (SBP) and diastolic blood pressure (DBP) parameters in patients treated with thrombolytic agents, reflecting those used in the National Institute of Neurological Disorders and Stroke rt‐PA (recombinant tissue plasminogen activator) Stroke Study. However, recent guidelines^1^, ^2^ have identified the limited evidence‐base supporting recommended acute ischemic stroke (AIS) blood pressure (BP) parameters. The National Institute of Neurological Disorders and Stroke rt‐PA study protocol recommended the use of manual sphygmomanometry due to concerns for bruising from automatic noninvasive oscillometric blood pressure (NIBP) monitors. Today, stroke practitioners throughout the world rely on NIBP device measures to guide treatment. Although NIBP devices are known to produce different values from manual sphygmomanometry,^6^ what constitutes clinically acceptable agreement between NIBP and manual methods has not been established.

Mean arterial pressure (MAP), or the average effective arterial pressure that propels blood through the vasculature, is recognized as the most valuable clinical parameter for assessment of tissue and organ perfusion.^7^, ^8^ In ischemic states, loss of autoregulatory capacity results in passive vasomotor dilation making blood flow passively dependent on MAP.^8^ Despite its significant effect on cerebral blood flow in the face of cerebral autoregulatory collapse,^8^ goal MAP parameters have never been set or even mentioned in stroke guidelines. In fact, although MAP is routinely displayed on NIBP devices, few interdisciplinary stroke clinicians make note of the measure or record the value in the clinical record.

Original research on the indirect oscillometric measurement of BP dates to 1876.^9^ NIBP devices determine MAP by measuring cuff pressure oscillations as the cuff pressure is reduced by discrete increments.^10^ In 1969, Posey et al and Geddes et al showed that the point of maximum oscillation corresponds to true MAP.^11^, ^12^ The small oscillations of intracuff pressure caused by heartbeat‐induced pulse volume changes are sensed by the cuff and measured by a pressure transducer.^13^ Cuff deflation in discrete increments allows the oscillation data obtained at each cuff pressure to be tested for artifacts and averaged, enhancing artifact‐rejection.^10^ The MAP is accepted as the most accurate measure produced by NIBP devices^14^ and is selected as the lowest cuff pressure at which the oscillation amplitude is a maximum.^10^ Both SBP and DBP values are algorithmically derived from the MAP^13^ and are not discretely measured by NIBP devices. Because cuff pressure oscillations continue when cuff pressure falls beneath DBP, the end point for diastolic pressure is considered indistinct.^15^

Few clinicians understand NIBP device performance. First, NIBP measurements are the end‐product of company‐owned proprietary computer algorithms that are not subject to independent critique and validation. Currently, there are thousands of unique automated devices manufactured by hundreds of companies,^16^ all with algorithms, testing, and validation procedures that differ from one device to another.^17^, ^18^ Because of this situation, claims and findings of accuracy and agreement for one NIBP device brand cannot be generalized to other brands. In fact, the best way to determine SBP and DBP from cuff pressure oscillations is considered an open scientific problem.^15^

Additionally, MAP is recognized as the only valid and reliable measure produced by NIBP devices^14^ when cardiac rhythm and contractile states remain constant. Because of this, NIBPs are not recommended for use in rhythms such as atrial fibrillation because MAP cannot be accurately measured by oscillometric means.^19^ Because NIBP cuffs deflate at a manufacturer‐specific speed that assumes a regular pulse,^20^ NIBP use in patients with irregular heart rhythms results in highly inaccurate BP readings.^19^ Third, study findings^6^, ^14^, ^15^, ^21^, ^22^, ^23^, ^24^, ^25^, ^26^, ^27^, ^28^, ^29^, ^30^ vary substantially with regard to agreement between NIBP and measures from an arterial line or manual sphygmomanometer, with high rates of disagreement noted at borderline hypertensive and higher BP levels.^6^

Because the alteplase BP parameters used today were derived from a study using manual sphygmomanometry, we sought to understand agreement between NIBP and manual measures for SBP, DBP, and MAP in this vulnerable patient cohort.

## Methods

The data that support the findings of this study are available from the corresponding author upon reasonable request and ethics board approval.

We conducted a multisite prospective observational study to assess agreement between manual sphygmomanometry‐derived and NIBP‐derived BP values during the 24‐hour observation period post‐thrombolytic administration for AIS. Patients were enrolled over 13 consecutive months.

Institutional review board approval with waiver of informed consent was granted. Study procedures did not interfere with standard of care treatment for patients with AIS; in particular, time to alteplase tPA bolus, initiation of the tPA drip, and provision of other routine care procedures were not interfered with, as the study focused solely on device agreement.

### Study Protocol

Patients with AIS who met inclusions without exclusions for alteplase treatment as detailed in American Stroke Association guidelines,^1^, ^2^ which were in sinus rhythm on ECG,^19^, ^31^ with upper arm circumference measures meeting cuff sizing recommendations,^32^ and not requiring any form of isolation or contact precautions were eligible for enrollment.

Our methods and interpretation of findings were directed by published standards for NIBP validation procedures^17^, ^33^, ^34^, ^35^ which requires paired measures from at least 85 subjects with the auscultatory standard retained for reference BP measurement and measures taken simultaneously by 2 trained observers blinded to each other's readings and to measures taken with the test device before obtaining their auscultatory measures; for analyses, the mean error was expected to fall within 5 mmHg and the SD of the error was expected to fall within 8 mmHg. All measurements were taken after completion of the alteplase infusion, within the 24‐hour period coinciding with stringent thrombolytic patient monitoring. All coinvestigators were trained to use the manual BP measurement technique described by Pickering,^32^ which was complimented by 2 investigators using a dual‐auditory stethoscope.

Two investigators at each study site measured 5 sets of manually derived and NIBP‐derived SBP, DBP, and MAP. All study devices (Drager NIBP; General Electric NIBP) had been approved for use by biomedical engineering and displayed current labels showing the monitoring time period for which they were approved. Both manual BP and NIBP measurements were taken from the same arm. Manual BPs were always taken before NIBP measures in accordance with the NIBP validation standards^17^, ^33^, ^34^, ^35^ to reduce the potential for bias in the 2 investigators performing manual sphygmomanometry. BP measures taken as a part of this study were not used to guide patient treatment per recommendation of the institutional review board. Arm circumference was measured and the appropriately sized cuff was selected for application with the arm placed level to the phlebostatic axis.^32^, ^36^ Prestige Medical Clinical I Teaching Edition dual‐auditory stethoscopes were used to ensure accuracy, with investigators blinded to each other's auscultatory pressure measures; disagreement of more than 4 mmHg between investigators prompted remeasurement of pressures after a specified 5‐minute waiting period following the same procedures as described. Once pressures were recorded, the manual cuff was removed, and the appropriately sized NIBP cuff was placed using standard of care technique. Each of the 2 measurement methods was taken within 5–8 minutes of each other to allow time for arm revascularization and relaxation between measurements according to evidence‐based recommendations for BP monitoring.^23^, ^38^

### Statistical Analysis

Data were entered into IBM SPSS Statistics Version 22.0 for descriptive analysis, tests of significance (parametric and nonparametric *t*‐test), and normality analyses. NCSS Statistical Software (2020)^37^ was used for Bland–Altman (B‐A) analyses for measures of agreement between sets of manually‐ and NIBP‐derived SBP, DBP, and MAP measures.^38^, ^39^, ^40^, ^41^ The specific research question consistent with B‐A methods^38^ was, “*Are manually derived and NIBP‐derived arterial blood pressures comparable to the extent that one might replace the other with sufficient accuracy for the purpose of precise blood pressure measurement in patients treated with thrombolytic agents*.” We incorporated B‐A plots to illustrate the magnitude of disagreement, spot outliers, and determine data trends with displays of 4 types of data misbehavior: (1) systematic error (mean offset), (2) proportional error (trend), (3) inconsistent variability, and (4) excessive or erratic variability.^42^ Shapiro‐Wilk tests of normality were performed, and if significant differences were documented, nonparametric 1‐sample Wilcoxon signed rank tests were performed to examine differences between groups. Measurement points were expected to center about difference=0, with a reasonable CI and maintenance of the same general pattern for all horizontal axis values.^42^ Erratic data variability was defined as greater than 5% of data falling outside the CI for each measure.^42^ Sets of measures were treated independently. Lins concordance correlation coefficient was computed to measure strength of agreement between values obtained by the 2 methods of BP measurement. Analyses also included device performance by manufacturer brand to determine if different devices performed similarly.

Lastly, we adopted the British Hypertension Society (BHS)^43^ and the European Society of Hypertension International Protocol (ESHIP)^34^ for evaluating NIBP device accuracy by documenting the percentage of readings that fall within the 5 and 10 mmHg difference of each other. Consistent with the BHS/ESHIP protocol, we also determined whether the mean differences fell within the Association for the Advancement of Medical Instrumentation recommendations of <5 and ≤8 mmHg, respectively. Measures were also evaluated against BHS/ESHIP guidelines that stipulate that 80% of measures should fall within 5 mmHg, and 95% should fall within 10 mmHg.^34^

## Results

A total of 7 hospitals (3 comprehensive stroke centers – 2 in New Jersey and 1 in Tennessee, and 4 primary stroke centers in New Jersey) served as the sites for this study. Between February 2019 and February 2020, through nonrandom convenience sampling, we enrolled 95 patients treated with alteplase with AIS: 62 from New Jersey and 33 from Tennessee; mean age 69±14; 61% men; 76% with a history of hypertension; there were no differences in BP values by sex (Table 1).

**Table 1:** Demographic/Baseline Characteristics of the Study Population

Variables	All study sites (n=95)	Tennessee values (n=33)	New Jersey values (n=62)	*P* value (*t*‐test, chi‐square, and median test)
Age, mean (SD)	69 (14)	65 (12.3)	71 (14.6)	<0.05* (0.04)
Sex				*P*=ns (0.41)
Male, n (%)	58 (61)	22 (67)	36 (58)	
Female, n (%)	37 (39)	11 (33)	26 (42)	
Ethnicity and race				<0.05** (0.000)
White, n (%)	61 (64)	9 (27)	52 (84)	
Black, n (%)	30 (32)	23 (70)	7 (11)	
Hispanic, n (%)	4 (4)	1 (3)	3 (5)	
Hypertension, n (%)	72 (76)	24 (73)	48 (77)	*P*=ns (0.61)
Diabetes , n (%)	33 (35)	14 (42)	19 (31)	*P*=ns (0.25)
HbA1c, mean (SD)	6.4 (1.6)	6.5 (1.8)	6.3 (1.4)	*P*=ns (0.58)
Smoking, n (%)	30 (32)	11 (33)	19 (31)	*P*=ns (0.79)
Previous stroke, n (%)	25 (27)	13 (41)	12 (19)	<0.05* (0.03)
Baseline NIHSS, median (Q1, Q3)	5 (3, 11)	5 (3, 7)	6 (3.25, 13)	<0.05* (0.04)
Prestroke modified Rankin score, median (Q1, Q3)	0 (0, 1)	0 (0, 2)	0 (0, 1)	*P*=ns (0.99)

*P*=ns refers to nonsignificant; ^*^ *P* value<0.05; ^**^ *P* value<0.001. For categorical variables (sex, ethnicity and race, hypertension, diabetes, smoking, previous stroke) chi‐square test was used. For continuous variables (age, A1C), a Student's *t*‐test was used. For baseline NIHSS and baseline mRS, nonparametric median test for difference in the medians. mRS indicates modified Rankin scale; and NIHSS, National Institutes of Health Stroke Scale.

### Bland–Altman Analyses

The Figure[Fig svi212755-fig-0001]presents B‐A and linear scatter plots for SBP, DBP, and MAP. Mean difference between NIBP and manual SBP was equal to −3.75 mmHg (SD=12.8) with a range in limits of agreement spanning −28.91 to 21.41 mmHg (normality rejected: Shapiro–Wilk *P*=0.002; 1 sample Wilcoxon signed rank *P*<0.001). Additionally, the higher the SBP, the greater the disagreement in measures. Lin's concordance correlation coefficient demonstrated poor agreement^44^ between measures at 0.8. Similarly, for DBP mean difference between NIBP and manual measures was −1.06 mmHg (SD=10.2) with a range in limits of agreement spanning −21.0 to 19.0 (normality rejected: Shapiro–Wilk *P*=0.027; 1 sample Wilcoxon signed rank *P*=0.049), and Lin's concordance correlation coefficient demonstrated poor agreement^44^ between measures at 0.69. For MAP, mean difference between methods was −5.49 mmHg (SD=11.2) with a range of limits of agreement spanning −27.5 to 16.5 (normality rejected on Shapiro–Wilk *P*=0.01; 1 sample Wilcoxon signed rank *P*<0.001) and Lin's concordance correlation coefficient demonstrated poor agreement^44^ between measures at 0.7. There was no difference in device agreement by manufacturer brand.

**Figure svi212755-fig-0001:**
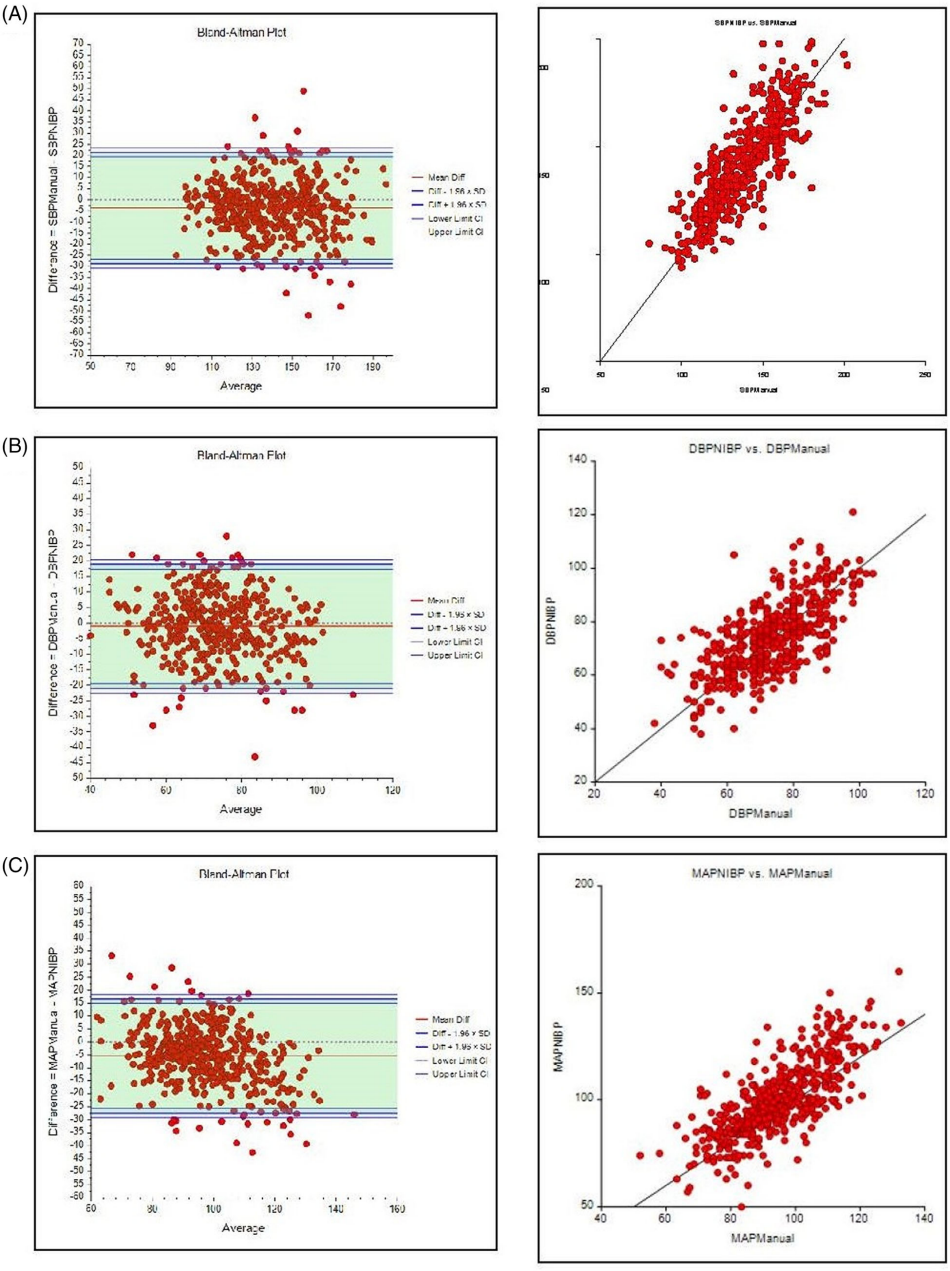
**Bland Altman (BA) and Linear Scatterplots for (A) SBP (B) DBP and (C) MAP**. **BA Plot for SBP**: The mean is offset, lying below zero, suggesting a mean bias of −3.75. The negative value indicates that the NIBP measures are higher than the manual measures. Most of the outliers fall beyond an average reading of approx. 130 mmHg SBP. The higher the SBP, the greater the disagreement. **BA Plot for DBP**: line of bias falls below the test value of zero, indicating that the diastolic NIBP measures are higher than manual measures. This difference of −1.06 is not as extreme as it was with SBP. Most of the outliers fall beyond an average DBP reading of approx. 45 mmHg. **BA Plot for MAP**: extreme differences for a calculated MAP derived from manual pressures vs the MAP derived from NIBP devices. The mean bias is approximately −5.5. The outliers occur almost across the entire spectrum. **Linear Scatter Plots for all 3 paired measures**: the data points are deviating (scattered) from the centerline of equality and are widely dispersed, indicating that the linear relationship is not strong. There is a difference in the manual and NIBP readings.

Table 2 presents the percentage of paired manual and NIBP measures falling within the BHS/ESHIP guidelines 5–10 mmHg limits, alongside the percentage of measures falling within 15 and 20 mmHg in the sample. For SBP, only 39% of paired measures fell within 5 mmHg of each other; a cumulative 62% fell within 10 mmHg, leaving 38% of measures outside the acceptable limits. For DBP, 44% of paired measures fell within 5 mmHg of each other, with a cumulative 72% falling within 10 mmHg, leaving 28% beyond acceptable limits. Lastly, for MAP 34% of paired measures fell within 5 mmHg of each other, a cumulative 62% fell within 10 mmHg, leaving 38% beyond acceptable limits.

**Table 2:** Percentage of Manual and NIBP Measures Falling Within 5–20 mmHg Difference of Each Other

Variable (n=475)	± ≤5 mmHg (%)	± ≤10 mmHg (%)	± ≤15 mmHg (%)	± ≤20 mmHg (%)	Mean±SD of the difference (mmHg)
SBP	39	62	76	86	−3.75±12.84
DBP	44	72	86	95	−1.06±10.28
MAP	34	62	78	89	−5.49±11.22

DBP indicates diastolic blood pressure; MAP, mean arterial pressure; NIBP, noninvasive oscillometric blood pressure; and SBP, systolic blood pressure.

## Discussion

Our study found significant disagreement between paired manual sphygmomanometer‐derived and NIBP‐derived measures for SBP, DBP, and MAP. Importantly, our results show that NIBP measures were most often higher than manual measurements and differed from manual measurements as much as 50 mmHg for SBP, 40 mmHg for DBP, and 44 mmHg for MAP. Using the BHS/ESHIP guidelines stipulating that 80% of measures should fall within 5 mmHg and 95% should fall within 10 mmHg^34^; we found that neither SBP, DBP, nor MAP held up to this standard. In fact, it was not until we examined the percent of measures falling within a 20 mmHg difference that DBP alone hit the 95% threshold, and arguably 20 mmHg is a clinically significant difference.

Clinically acceptable limits of agreement between devices measuring SBP, DBP, and MAP in patients with AIS reperfusion should be defined a priori; however, substitution of NIBP for manual sphygmomanometer‐derived arterial pressure is common today, and few clinicians consider the implications of a lack in measurement agreement between these devices. Interestingly, standards for comparing device agreement recommend that they are not intended to replace responsible clinical decision‐making,^17^ yet no clinical expectations have been set to address how users should routinely compare differences between measures and under what circumstances clinicians should reject use of a device because of considerable differences in level of agreement. A related problem in determination of agreement is that algorithms, testing, and validation procedures differ from one device to another^17^ and are scattered in many different publications or patents,^18^ making claims and agreement findings for one NIBP brand incomparable to other brands. The accurate extraction of systolic and diastolic pressure oscillations is considered an open problem in biomedical engineering.^15^

Level of agreement estimates the interval within which a proportion of the differences between measurements lie and includes both systematic (bias) and random error (precision).^45^ However, stroke guidelines are silent regarding acceptable error in arterial pressure measurement between devices, and because errors should ideally be absent when assessing patients with reperfusion AIS, we, therefore, compared our findings to a test value of 0. Although admittedly, this is an ideal level of agreement, an argument can be made that in practice, measures of arterial pressure should not differ more than at most 3–5 mmHg between devices, and this is especially important given that clinical management of arterial pressure is an important aspect of reperfusion treatment. B‐A analyses only define intervals of agreement and do not determine whether those limits are acceptable (or not) from a clinical standpoint. How far apart measurements can be to maintain their acceptability in clinical use is a question of judgment that must be considered cautiously.^46^ We argue that determination of acceptable levels of agreement should ideally be made in all vascular disease conditions and be used to guide how devices are tested and used in clinical settings based on biological considerations and treatment goals.^47^ Our findings highlight this need, particularly because NIBP is almost exclusively used today, yet the BP goals for treatment with alteplase were originally established to support BP measurement by manual cuff.

Our methods ensured a highly evidence‐based measurement approach that included such things as measurement of arm circumference with selection of appropriate cuff size, ensuring the arm was maintained at the phlebostatic axis, excluding patients with atrial fibrillation or other dysrhythmias, allowing the recommended 5–8 minutes revascularization pause between measurement, and using dual auscultatory measurement. When comparing our methods to routine clinical application of NIBP devices, the risk of error is likely significantly higher during routine use where attention to these details is often lacking. Because MAP is used by NIBP devices to algorithmically derive SBP and DBP,^13^, ^14^ the lack of a MAP goal in acute stroke patient management is concerning, especially since calculated MAPs differ considerably from NIBP‐derived MAP measures challenging retrospective work that has attempted to calculate MAP from NIBP‐derived SBP and DBP.^48^ Additionally, although the calculated MAP from 175/100 mmHg is 125 mmHg, a similar MAP can correspond to SBP and/or DBP limits that are outside guideline boundaries for patients treated with systemic thrombolysis (eg, 215/80 or 160/107 mmHg).

Our findings are consistent with others that have found significant differences between device measures, with oscillometric NIBP devices overestimating especially SBP, compared with sphygmomanometry.^6^, ^28^, ^29^, ^30^ Therefore, it is very possible that NIBP use results in greater antihypertensive treatment in patients treated with alteplase, which may produce much lower arterial pressures than recognized on an NIBP device, especially when compared with traditional BP lowering recommendations in AIS.^2^, ^3^, ^4^, ^5^, ^49^

Our study has limitations that must be acknowledged. First, we did not obtain continuous measures over the 24‐hour period, however, continuous BP measurement was unnecessary to meet our study objective informed by guidelines.^17^, ^33^, ^34^, ^35^, ^43^ Therefore, we argue that our methods were acceptable. We also could not test every brand of marketed NIBP device in use in clinical practice. However, our findings reflect performance of 2 commonly used NIBP devices and therefore are likely generalizable. Lastly, although arterial line measurement is commonly considered the “gold standard” for determination of “true” SBP, DBP, and MAP values, we chose to use the methods established in the original National Institute of Neurological Disorders and Stroke rt‐PA Stroke Study,^49^ which relied on manual sphygomomanometry as the point of comparison with NIBP since the BP parameters that originated with this study are still in use today. Additionally, placement of an arterial line in patients immediately post‐alteplase would constitute an unnecessary invasive procedure that does not reflect standard of care, and insertion before thrombolytic administration would have delayed treatment.

## Conclusion

In their 2019 scientific statement, the American Heart Association wrote that there is little, if any, evidence available on the validation of BP measurements obtained in the acute care setting.^50^ Despite this, acute stroke practitioners rely on NIBP devices to guide treatment of patients with AIS. Our results show that NIBP measures are consistently higher than, and may differ considerably from, manual measurements and safe MAP limits for patients with thrombolytic‐treated AIS remains unknown.^50^ Future work must be guided by consensus for acceptable levels of BP device agreement. Our study takes an important first step in reframing the AIS treatment context toward consideration of MAP as a key measure to guide the initiation of thrombolytic treatment and ongoing patient management.

## Author Contributions

Mani Paliwal, MS, MBA – Hackensack Meridian Health, Edison, NJ. Anne Shearin – University of Tennessee College of Nursing, Memphis, TN. Jane Kaiser, MSN – Ocean University Medical Center, Brick, NJ. Eun Sun Koo – Jersey Shore University Medical Center, Neptune, NJ. Danielle Howey – Ocean University Medical Center, Brick, NJ. Michele Galati – Southern Ocean Medical Center, Manahawkin, NJ. Bozena Czekalski, MSN – Riverview Medical Center, Red Bank, NJ. Jennifer Dumawal, DNP – Bayshore Medical Center, Holmdel, NJ. Briana DeCarvalho, MSN – JFK Medical Center, Edison, NJ. Jackie Dwyer, MSN – Jersey Shore University Medical Center, Neptune, NJ. Georgios Tsivgoulis, MD, PhD – National and Kapodistrian University of Athens, Greece. Andrei V. Alexandrov, MD – Banner University Medical Center and University of Arizona, Phoenix. Anne W. Alexandrov, PhD, FAAN – University of Tennessee Health Science Center, Memphis, TN.

## Sources of Funding

None.

## Disclosures

None.
